# Sequence Analyses and Phenotypic Characterization Revealed Multidrug Resistant Gene Insertions in the Genomic Region Encompassing Phase 2 Flagellin Encoding *fljAB* Genes in Monophasic Variant *Salmonella enterica* Serovar 4,5,12:i:- Isolates From Various Sources in Thailand

**DOI:** 10.3389/fmicb.2021.720604

**Published:** 2021-10-04

**Authors:** Aye Thida Win, Sirirak Supa-amornkul, Renato H. Orsi, Jaclyn H. Carey, William J. Wolfgang, Soraya Chaturongakul

**Affiliations:** ^1^Department of Microbiology, Faculty of Science, Mahidol University, Bangkok, Thailand; ^2^Mahidol International Dental School, Faculty of Dentistry, Mahidol University, Bangkok, Thailand; ^3^Department of Food Science, Cornell University, Ithaca, NY, United States; ^4^Bacteriology Laboratory, New York State Department of Health, Wadsworth Center, Albany, NY, United States; ^5^Center of Microbial Genomics (CENMIG), Faculty of Science, Mahidol University, Bangkok, Thailand

**Keywords:** monophasic *S*. Typhimurium, *fljAB* region, antimicrobial resistance, WGS—whole-genome sequencing, STM2759 and *iroB*

## Abstract

*Salmonella enterica* serovar 4,5,12:i:- (*S*. 4,5,12:i:-), a monophasic variant of *Salmonella* Typhimurium (STm) lacking the phase 2 flagellin encoding genes *fljAB*, has become increasingly prevalent worldwide. The increasing trends in multidrug resistant (MDR) *S*. 4,5,12:i:- prevalence also pose an important global health threat. Though many reports have characterized phenotypic and genotypic drug resistance of this serovar, few studies have characterized antimicrobial resistance of this serovar in Thailand. In this study, 108 *S*. 4,5,12:i:- isolates from various sources in Thailand and four international *S*. 4,5,12:i:- isolates were screened using polymerase chain reaction (PCR) to detect the presence of five target regions which are associated with antimicrobial resistant (AMR) genes, in the genomic region that contained *fljAB* genes in STm. We determined AMR phenotypes of all isolates by Kirby-Bauer disk diffusion method. Whole genome sequencing (WGS) was performed on 53 representative isolates (based on differences in the pulsed filed gel electrophoresis profiles, the sources of isolate, and the PCR and AMR patterns) to characterize the genetic basis of AMR phenotype and to identify the location of AMR determinants. Based on PCR screening, nine PCR profiles showing distinct deletion patterns of the five target regions have been observed. Approximately 76% of isolates (or 85 of 112 isolates), all of which were Thai isolates, contained five target regions inserted between STM2759 and *iroB* gene. A total of 21 phenotypic AMR patterns were identified with the predominant AmpST resistant phenotype [i.e., 84% (or 94 of 112) tested positive for resistance to ampicillin, streptomycin, and tetracycline], and 89% (or 100 of 112) were found to be MDR (defined here as resistant to at least three classes of tested antimicrobials). Using WGS data, a total of 24 genotypic AMR determinants belonging to seven different antimicrobial groups were found. AMR determinants (i.e., *bla_TEM__–__1_*, *strB-A*, *sul2*, and *tetB*, conferring resistance to ampicillin, streptomycin, sulfonamides, and tetracycline, respectively) were found to be inserted in a region typically occupied by the phase 2 flagellin encoding genes in STm. These resistant genes were flanked by a number of insertion sequences (IS), and co-localized with mercury tolerance genes. Our findings identify AMR genes, possibly associated with multiple IS*26* copies, in the genetic region between STM2759 and *iroB* genes replacing phase 2 flagellin encoding *fljAB* genes in Thai *S*. 4,5,12:i:- isolates.

## Introduction

*Salmonella enterica* is a diverse Gram-negative zoonotic bacterium globally recognized as a cause of epidemic foodborne outbreaks. Of all foodborne diseases caused by various pathogens, diarrhea and invasive salmonellosis due to non-typhoidal *Salmonella* (NTS) serovars are affecting tens of millions of people worldwide annually, with more than hundred thousand deaths (Havelaar et al., 2015; Kirk et al., 2015). A large number of animals, particularly food animals, have been identified as reservoirs or sources for NTS serovars and are responsible for numerous human outbreaks through consumption of contaminated meat (Bangtrakulnonth et al., 2004; Hoelzer et al., 2011). The emerging monophasic variant *S. enterica* serovar 4,5,12:i:- (thereafter referred as *S*. 4,5,12:i:-), regarded as one of the derivatives of *S*. Typhimurium (STm), which has the antigenic formula 4,5,12:i:1,2, has been substantially increasing in prevalence across many regions around the world (European Food Safety Authority [EFSA], 2008; Centers for Disease Control and Prevention [CDC], 2011; European Food Safety Authority [EFSA] and European Centre for Disease Prevention and Control [ECDC], 2016; Biswas et al., 2019; Sun et al., 2020). In Thailand, *S*. 4,5,12:i:- has been among the top five most commonly reported *Salmonella* serovars during the last two decades (Bangtrakulnonth et al., 2004; Thailand Department of Medical Sciences, 2016; Patchanee et al., 2020; Luk-in et al., 2021). It is isolated from several kinds of animal sources, but the tendency of transmission among swine population is relatively high (de la Torre et al., 2003; Mossong et al., 2007; Hauser et al., 2010).

Previous investigations indicated that *S*. 4,5,12:i:- exhibited similar pathogenicity and virulence traits, along with the equivalent level of induction of host immune response as STm, consequently enhancing infectivity and dissemination patterns (Ikeda et al., 2001; Zeng et al., 2003; Parsons et al., 2013; Crayford et al., 2014). Though most of the phenotypic and genotypic characteristics of *S*. 4,5,12:i:- seem to be comparable to those of STm, a prominent difference is the lack of the phase 2 flagellar antigen encoding gene region, i.e., *fljAB* operon, in *S*. 4,5,12:i:- (Bugarel et al., 2012). Multiple distinct clones of *S*. 4,5,12:i:- have been reported to have emerged through various deletion events in the genomic region STM2759-*iroB* containing phase 2 flagellin encoding *fljAB* operon and surrounding genes (Garaizar et al., 2002; Soyer et al., 2009). In recent finding among food isolates of *S*. 4,5,12:i:- serovar from China (Yang et al., 2015), deletion patterns contributing to absence of *fljAB* genes with conserved *hin* and *iroB* genes were distinct from those of previously reported for Spanish, U.S. (Soyer et al., 2009), and Italian *S*. 4,5,12:i:- (Lucarelli et al., 2012) strains. Japanese and Malaysian clones have also been reported with varying deletion patterns between STM2757 and *hin* region (Ido et al., 2014). The study of *S*. 4,5,12:i:- isolates from Thailand by Huoy et al. (2014) revealed that deletion patterns in the *fljAB* and *hin* genes containing regions have diverged from two international lineages, the U.S. and Spanish monophasic clones, suggesting the independent emergence. The evolutionary benefits of *fljAB*, *hin* and *iroB* gene deletions could be the gain fitness genes such as resistant genes and metal tolerance genes. In particular, Biswas et al. (2019) has demonstrated that *S*. 4,5,12:i:- sequence type (ST) 34 has gained its prevalence in place of ST19 in the past two decades. One of the unique characteristics of ST34 strains is that they contain genomic islands interspersed with inverted repeats flanking the above-mentioned fitness genes (García et al., 2016; Biswas et al., 2019; Branchu et al., 2019).

Although disease severity of salmonellosis ranges from mild diarrhea to severe life-threatening invasive infections (Jones et al., 2008; Crump et al., 2015), most of NTS infections are self-limiting in nature and treatment with antibiotic are not routinely necessary. However, the growing problem of the widespread emergence of multidrug resistant (MDR, defined as resistant to at least three classes of antimicrobials) *Salmonella* serovars is a major public health concern (Magiorakos et al., 2012). Among multiple *S.* 4,5,12:i:- clones emerged from different regions of the world, distinct antimicrobial resistance (AMR) patterns have been identified such as the tetra-resistant AMR pattern of AmpSSuT representing resistance to ampicillin, streptomycin, sulfonamide, and tetracycline which is regarded as the typical antibiotic resistant type (or R-type) of the European clones circulating in many European countries such as Italy, Germany, and the United Kingdom (Dionisi et al., 2009; Hauser et al., 2010; Hopkins et al., 2010; Lucarelli et al., 2010). Spanish clone exhibits resistance to multiple drugs with AmpCSuGSTSxT AMR pattern (resistant to ampicillin, chloramphenicol, sulfonamide, gentamicin, streptomycin, tetracycline, and co-trimoxazole) (Soyer et al., 2009; Laorden et al., 2010). In contrast to these MDR lineages, the U.S. clone is pan-susceptible to commonly used antimicrobials for *Salmonella* infections (Soyer et al., 2009). Among Thai *S*. 4,5,12:i: isolates over 90% were MDR, and a majority of the tested isolates showed AmpST resistance pattern (resistant to ampicillin, streptomycin, and tetracycline) (Huoy et al., 2014).

Continuous AMR surveillance is an essential in foodborne pathogens. The investigation is not only to monitor the effectiveness of current medical treatment but also to implement new preventive strategies against the emergence of multi-resistant strains (Oniciuc et al., 2018). Genomic analysis of *S*. 4,5,12:i:- European clones with resistant phenotype (R-Type) AmpSSuT by García et al. (2016) revealed resistance region 3 (RR3) locating between the STM2759*-iroB* loci and replacing the *fljAB* operon and *hin* gene. This RR3 was characterized by PCR targeting five regions designated as 131L, *tniA*, *tetC*Δ, MAK, and 131R to cover the entire *fljAB* deletion region (García et al., 2016). The genes within this RR3 comprised of those responsible for resistance to ampicillin, streptomycin, sulfonamides, tetracycline, and mercury. Recent report from Japan highlighted that majority of *S*. 4,5,12:i:- isolates obtained from various sources over 40 years (1976–2017) showed the presence of chromosomal *Salmonella* RR3 and composite transposons carrying antibiotic resistance genes *blaT_EM__–1B_*, *strA*, *strB*, *sul2*, and *tetB*, which is identical to the European *S*. 4,5,12:i:- clone (Arai et al., 2018).

Given similarities in the STM2759 and *iroB* region between European and Thai clones (Huoy et al., 2014), we hypothesized that this genetic region in Thai *S*. 4,5,12:i:- isolates contained insertions of antimicrobial resistant genes reflecting the phenotypic antimicrobial resistant profiles in Thai *S*. 4,5,12:i:- isolates. The objectives of this study were to (i) screen and compare the five target regions (131L, *tniA*, *tetC*Δ, MAK, and 131R) inserting between the two STM2759 and *iroB* loci, among the Thai and four international *S*. 4,5,12:i:- isolates, (ii) assess the phenotypic antimicrobial susceptibility patterns, and (iii) decipher the genetic basis of AMR in representative Thai *S*. 4,5,12:i:- isolates by whole genome sequencing.

## Materials and Methods

### Bacterial Strains Used in the Study

One hundred and eight bacterial isolates, previously serologically determined as *S.* 4,5,12:i:-, based on the White–Kauffmann–Le Minor scheme, were included in this study. Serotyping was confirmed with PCR, displaying typical loss of genes (*fljAB* and *hin* genes) required for expression of phase 2 flagellar antigen. Isolates were obtained from various sources in Thailand during a period between 2009 and 2012. Four additional monophasic *S.* 4,5,12:i:- isolates, two of each from Spain and the U.S., were acquired from Cornell University Food Safety Laboratory (CUFSL). Out of total 112 isolates, 66 have known PFGE patterns obtained from previous study (Huoy et al., 2014). All isolates were retrieved from animal-husbandry associated environment (7 isolates), food-producing animals (30 isolates), various categories of food products (33 isolates) including frozen raw meat, frozen ready-to-eat, fresh pork, as well as from human clinical cases (40 isolates), and from unknown sources (2 isolates). The metadata of the study isolates are shown in Supplementary Table 1.

### Polymerase Chain Reaction-Based Screening for the Presence of Five Target Regions (131L, *tniA*, *tetC*Δ, MAK, and 131R)

Screening for the presence of five intergenic regions locating between two genetic loci, STM2759 and *iroB* genes, was performed using conventional polymerase chain reaction (PCR) based on previous protocol (García et al., 2016) with a few modifications. After an overnight incubation at 37°C, a single colony from tryptic soy agar (TSA) (Becton, Dickinson and Company, United States) was inoculated in tryptic soy broth (TSB) (Becton, Dickinson and Company, United States), and incubated in shaker incubator at 37°C with shaking 200 rpm for 16–18 h. Extraction of genomic DNA from overnight cultured bacterial suspension was done using Presto^TM^ Mini gDNA Bacteria kit (Geneaid Biotech, Taiwan) according to the protocol recommended by manufacturers. Concentration of each purified DNA sample was measured using a NanoDrop spectrophotometer (Denovix, United States).

Approximately 20 ng/μl of DNA from each isolate was subjected for screening of the expected five target regions using the specific forward and reverse primers, targeting the left and right junctions (131L and 131R, respectively) of deleted region affecting the absence of phase 2 flagellin genes, and three internal regions (*tniA*, *tetC*Δ, and MAK) based on a previous study (García et al., 2016). A detailed description of primers used in this study is shown in Supplementary Table 2. After optimization of PCR conditions, we amplified five target regions of the 112 isolates with the use of GoTaq^®^ Green master mix (Promega, Madison, United States) and conventional PCR. For each reaction, a total of final reaction volume per reaction was 10 μl, consisting of GoTaq^®^ Green master mix (5 μl), 0.4 μM of each forward and reverse primer for each target region, DNA template of each isolate (1 μl) and DNAase-free distilled water. The PCR running conditions for each reaction were as follow: denaturation at 95°C × 5 min, 33 cycles of 95°C × 30 s, annealing for 131 L, *tetC*Δ, and MAK at 55°C × 30 s or for *tniA* and 131R at 60°C × 30 s, extension at 72°C × 3 min followed by final extension with 72°C × 10 min. Because of the absence of positive controls for five target regions, we considered the isolates as positive for having the genes based on their respective amplicon sizes (bp) of the amplified gene products that have already been known as shown in Supplementary Table 2. The amplified PCR products were visualized after running gel electrophoresis in 1% agarose gel (Vivantis Inc., United States) at 100 V for 60 min, and staining with 1X SYBR gold dye (10,000X) (Invitrogen, United States) for 40 min with shaking on platform shaker. GeneRuler^TM^ 1 kb plus DNA ladder (Fermentas, United States) was used as molecular size marker.

### Determination of Phenotypic Antimicrobial Susceptibility Patterns of *S*. 4,5,12:i:- Isolates

To determine *in vitro* antimicrobial susceptible properties of 112 study isolates, we performed Kirby-Bauer disk diffusion test in accordance with the standard procedure recommended by the Clinical and Laboratory Standards Institute (CLSI) (Clinical and Laboratory Standards Institute [CLSI], 2018 and Clinical and Laboratory Standards Institute [CLSI], 2020) using a panel of 10 standard antimicrobial drugs commonly used for antimicrobial susceptibility testing of *Salmonella* species. The standard antimicrobials with their respective concentrations are as follow: Amoxicillin/Clavulanic acid (Amc) 20/10 μg; Ampicillin (Amp) 10 μg; Cefotaxime (Ctx) 30 μg; Chloramphenicol (C) 30 μg; Ciprofloxacin (Cp) 5 μg; Nalidixic Acid (NA) 30 μg; Norfloxacin (Nor) 10 μg; Streptomycin (S) 10 μg; Tetracycline (T) 30 μg; Sulfamethoxazole/Trimethoprim (SxT) 23.75/1.25 μg (Clinical and Laboratory Standards Institute [CLSI], 2018, 2020). After overnight incubation, diameter of the zone of complete inhibition was measured. The isolates were interpreted as susceptible, resistant, or intermediate according to interpretative criteria based on CLSI breakpoints (Clinical and Laboratory Standards Institute [CLSI], 2018, 2020). Internal quality control was also assessed by using three control strains, namely *Escherichia coli* ATCC 25922, *Pseudomonas aeruginosa* ATCC 27853, and *Staphylococcus aureus* ATCC 25923.

### Whole Genome Sequencing of Representative Study Isolates

WGS was performed on selected Thai *S.* 4,5,12:i:- isolates. Selection criteria were based on: (1) the isolates having different PFGE profiles; (2) those having the same PFGE profile but from different sources; and (3) those having the same PFGE profile but with different PCR patterns and different antimicrobial resistance patterns obtained from previous steps. According to these criteria, 53 Thai isolates were selected for further investigation (Supplementary Tables 3, 4). DNA extraction and purification were performed using QIAamp^®^ DNA Micro Kit (Qiagen, Germany) in accordance with the manufacturer’s manual for Gram-negative bacteria. The quality and the quantity of extracted genomic DNA (gDNA) were assessed by a NanoDrop spectrophotometer (Denovix, United States). Extracted DNA was stored in −80°C freezer until sequencing.

Purified extracted gDNAs of 53 Thai *S*. 4,5,12:i:- isolates were sent to the Advanced Genomic Technologies Cluster New York State Department of Health/Wadsworth Center, NY (NYDOH) (31 non-human isolates) and Qiagen Company (Singapore) (22 human isolates) for WGS using Illumina platform. Pooled samples were whole-genome sequenced using the Illumina NextSeq500 instrument, using the Nextera XT library preparation protocol and the NextSeq^TM^ 500/550 Mid Output Kit v2 (2 × 150 paired-end) (NYDOH) and Illumina MiSeq instrument using the QIAseq FX DNA library preparation protocol and the Reagent Kit v2 (Qiagen). To assess the quality of raw reads, all sequencing reads were analyzed using FastQC v0.11.4 software, and raw reads were trimmed using the program trimmomatic v. 0.36. All sequence reads with the average phred quality score above 30 were included in further analysis. Genome assembly of all trimmed sequencing reads was performed using CLC Genomics Workbench v11.0.1. Sequences were aligned and mapped using the programs BBmap v37.50 and Samtools v. 1.8 against *S.* 4,5,12:i:- strain S04698-09 (ENA ref LN999997.1) (Elnekave et al., 2018; Mastrorilli et al., 2018) to compute coverage statistics. *In silico* multilocus sequence typing (MLST) was performed using nucleotide sequences of seven house-keeping gene alleles from the draft assemblies and matched with the profiles from MLST database to identify the associated MLST using MLST v1.8^1^ (Thomsen et al., 2016; Mastrorilli et al., 2018). Serovar identification by traditional serotyping was confirmed by using SeqSero v1.2 software through sequencing data.

### Bioinformatic Analysis of Genome Sequencing Data

To identify AMR genes, assembled data of each strain were subjected for identification of AMR genes using ResFinder v3.0^2^ implemented in the Bacterial Analysis Pipeline^3^ with the following options: the threshold for reporting a match between a gene in the database and the input genome was set to be 80% identity with at least 60% coverage of the length of the acquired resistance gene sequence.

To assess the location of AMR genes particularly in genomic regions of five targets between STM2759 and *iroB* genes or outside this specific region, assembled contigs were subjected to functional annotation using an automated web-based tool Rapid Annotation using Subsystem Technology v2.0 (RAST)^4^ (Aziz et al., 2008) to produce high-quality annotation of the assembly. To compare our samples with reference genomes already established in public database, complete genome sequence of STm LT2 (GenBank: accession number AE006468.2), finished genome sequence of *S*. 4,5,12:i:- strain S04698-09 (ENA ref LN999997.1), and reference genome sequence of resistance region (RR3) in *S*. 4,5,12:i:- strain 07-2006 (GenBank: accession number KR856283) from the previous study (García et al., 2016) were also annotated. Annotated data from all selected Thai strains and complete genome sequences of the two references were viewed and compared with Seed Viewer v2.0^5^ (Overbeek et al., 2014). Further verification and identification of gene contents and genetic structure in the specific region between STM2759 and *iroB* genes were conducted. The loci of resistance genes responsible for phenotypic AMR patterns within or outside the interested region were assessed.

## Results

### The Five Target Regions Between STM2759 and *iroB* Genes Were Present in 75.9% Thai *S.* 4,5,12:i:-

PCR-based identification of the five target regions (131L, *tniA*, *tetC*Δ, MAK, and 131R) spanning from STM2759 to STM2773 (*iroB*) among 108 Thai *S*. 4,5,12:i:- isolates and four non-Thai isolates (two Spanish and two U.S. strains) was performed. We identified nine patterns of presence or absence for the five regions (patterns 1–9 as shown in Table 1). Most of the tested isolates (85 of 112 isolates, 75.9%) had all five target regions (PCR pattern 1). Unlike Thai isolates, one Spanish and both U.S. isolates were negative for all five target regions (PCR pattern 9). The second most common PCR pattern found in this study (17 isolates or 15.2%) was PCR pattern 2 (missing only the 131L target region). The remaining PCR patterns were less likely to be identified among the study isolates (approximately 1–2%). The leftmost region (131L) was found to be the most commonly absent region, being undetectable in 25 isolates (22.3%) including the four non-Thai *S*. 4,5,12:i:- strains.

**TABLE 1:** PCR patterns showing the presence of five target regions identified in 112 isolates of *S*. 4,5,12:i:- related to geographical sources (Thailand, Spain, and the U.S.).

**PCR profile of the 5 target regions**						**Number of isolates (%)**			
**131L**	***tniA***	***tetC*Δ**	**MAK**	**131R**	**PCR pattern**	**Thailand**	**Spain**	**US**	**Total**
+	+	+	+	+	**1**	85 (75.9)	0	0	85 (75.9)
−	+	+	+	+	**2**	17 (15.1)	0	0	17 (15.1)
−	−	+	+	+	**3**	1 (0.9)	0	0	1 (0.9)
−	+	+	+	−	**4**	2 (1.8)	0	0	2 (1.8)
+	+	+	+	−	**5**	1 (0.9)	0	0	1 (0.9)
+	−	+	+	−	**6**	1 (0.9)	0	0	1 (0.9)
−	−	−	+	−	**7**	1 (0.9)	0	0	1 (0.9)
−	−	+	−	−	**8**	0	1 (0.9)	0	1 (0.9)
−	−	−	−	−	**9**	0	1 (0.9)	2 (1.8)	3 (2.7)
Total						**108 (96.4)**	**2 (1.8)**	**2 (1.8)**	**112 (100)**

### Polymerase Chain Reaction Patterns Positive for the Five Target Regions Can Be Used as Indicators of AmpST Multidrug Resistant

To investigate the AMR phenotypes of the *S.* 4,5,12:i:- isolates, disk diffusion assays were performed. A total of 21 phenotypic AMR patterns (patterns 1–21) were observed among tested isolates (Table 2). Out of 21 AMR patterns, 19 patterns were present among Thai isolates. Almost all isolates (111 of 112 isolates, 99%) were resistant to at least one drug, and 100 isolates (89%) were found to be MDR i.e., resistant to at least three antimicrobial classes. The maximum number of drugs resisted by tested isolates was eight (AMR pattern 1: AmpCtxCCpNASTSxT) which was detected only in one Thai isolate while AMR pattern 21 (sensitive to all tested drugs) was found in two Thai isolates (2%). The most common AMR pattern detected in this study was pattern 14, AmpST (resistant to ampicillin, streptomycin, and tetracycline), comprising 52% of tested isolates (59 of 112 isolates) and was found only in Thai isolates. The next most common patterns were 12 (AmpT) and 16 (AmpS), representing about 6 and 5%, respectively. The remaining AMR patterns were less frequently observed, accounting for 1–5 isolates in each AMR pattern. The most common PCR patterns observed in this study, PCR patterns 1 and 2, were found to be related with the most common AMR pattern obtained from this study which is AMR pattern 14 (AmpST) (Table 3).

**TABLE 2:** AMR patterns found in the study *S.* 4,5,12:i:- isolates (*n* = 112).

AMR profile*	**AMR Pattern**	**Number of isolates (%)**			
		**Thailand**	**Spain**	**US**	**Total**
AmpCtxCCpNASTSxT	**1**	1 (0.9)	−	−	1 (0.9)
AmpCtxCCpSTSxT	**2**	3 (2.7)	−	−	3 (2.7)
AmpCtxCCpST	**3**	5 (4.5)	−	−	5 (4.5)
AmpCCpSTSxT	**4**	5 (4.5)	−	−	5 (4.5)
AmpCtxCpST	**5**	1 (0.9)	−	−	1 (0.9)
AmpCSTSxT	**6**	4 (3.6)	1 (0.9)	−	5 (4.5)
AmpCCpST	**7**	1 (0.9)	−	−	1 (0.9)
AmcAmpCpS	**8**	1 (0.9)	−	−	1 (0.9)
AmcAmpST	**9**	1 (0.9)	−	−	1 (0.9)
AmpCtxST	**10**	1 (0.9)	−	−	1 (0.9)
AmpCST	**11**	5 (4.5)	−	−	5 (4.5)
AmpCpST	**12**	7 (6.3)	−	−	7 (6.3)
AmpCpS	**13**	4 (3.6)	−	−	4 (3.6)
AmpST	**14**	59 (52.7)	−	−	59 (52.7)
CST	**15**	−	1 (0.9)	−	1 (0.9)
AmpS	**16**	6 (5.4)	−	−	6 (5.4)
CpS	**17**	−	−	1 (0.9)	1 (0.9)
CpT	**18**	1 (0.9)	−	−	1 (0.9)
ST	**19**	1 (0.9)	−	−	1 (0.9)
S	**20**	1 (0.9)	−	1 (0.9)	2 (1.8)
All sensitive	**21**	1 (0.9)	−	−	1 (0.9)
Total		108	2	2	**112**

*AMR profile represents the isolates showing resistant to the tested drugs. The isolates presenting intermediate resistance were considered as resistant. Amp, Ampicillin; Amc, Amoxycillin/Clavulanic acid; Ctx, Cefotaxime; C, Chloramphenicol; Cp, Ciprofloxacin; NA, Nalidixic acid; S, Streptomycin; T, Tetracycline; SxT, Sulfamethoxazole/Trimethoprim.

**TABLE 3:** AMR patterns in relation to PCR patterns in *S*. 4,5,12:i:- isolates (*n* = 112).

**PCR pattern**		**1**	**2**	**3**	**4**	**5**	**6**	**7**	**8**	**9**	**Total**
		
AMR pattern*		**+++++**	−**++++**	−−**+++**	−**+++**−	**++++**−	**+**−**++**−	−−−**+**−	−−**+**−−	−−−−−	
AmpCtxCCpNASTSxT	**1**	0	1	0	0	0	0	0	0	0	1
AmpCtxCCpSTSxT	**2**	3	0	0	0	0	0	0	0	0	3
AmpCtxCCpST	**3**	1	2	0	2	0	0	0	0	0	5
AmpCCpSTSxT	**4**	5	0	0	0	0	0	0	0	0	5
AmpCtxCpST	**5**	1	0	0	0	0	0	0	0	0	1
AmpCSTSxT	**6**	4	0	0	0	0	0	0	0	1	5
AmpCCpST	**7**	0	1	0	0	0	0	0	0	0	1
AmcAmpCpS	**8**	0	1	0	0	0	0	0	0	0	1
AmcAmpST	**9**	1	0	0	0	0	0	0	0	0	1
AmpCtxST	**10**	1	0	0	0	0	0	0	0	0	1
AmpCST	**11**	1	4	0	0	0	0	0	0	0	5
AmpCpST	**12**	7	0	0	0	0	0	0	0	0	7
AmpCpS	**13**	1	1	0	0	1	0	1	0	0	4
AmpST	**14**	56	2	1	0	0	0	0	0	0	59
CST	**15**	0	0	0	0	0	0	0	1	0	1
AmpS	**16**	4	1	0	0	0	1	0	0	0	6
CpS	**17**	0	0	0	0	0	0	0	0	1	1
CpT	**18**	0	1	0	0	0	0	0	0	0	1
ST	**19**	0	1	0	0	0	0	0	0	0	1
S	**20**	0	1	0	0	0	0	0	0	1	2
All sensitive	**21**	0	1	0	0	0	0	0	0	0	1
Total		85	17	1	2	1	1	1	1	3	**112**

PCR patterns related to presence/absence of the 5 target regions (131L, *tniA*, *tetC*Δ, MAK, and 131R). *AMR profile represents the isolates showing resistant to the tested drugs. The isolates presenting intermediate resistance were considered as resistant. Amp, Ampicillin; Amc, Amoxycillin/Clavulanic acid; Ctx, Cefotaxime; C, Chloramphenicol; Cp, Ciprofloxacin; NA, Nalidixic acid; S, Streptomycin; T, Tetracycline; SxT, Sulfamethoxazole/Trimethoprim.

### Whole Genome Sequencing Analyses of Representative *S*. 4,5,12:i:- Isolates Confirmed Polymerase Chain Reaction Patterns of the Five Target Regions and Multidrug Resistant Phenotypes

In order to confirm the presence of the five target regions and the gene contents between STM2759 and *iroB* and to locate all antimicrobial resistant genes, WGS was performed in 53 selected Thai *S*. 4,5,12:i:- isolates, representing different sources, molecular subtypes (PFGE patterns), and PCR patterns of five main regions and AMR profiles. Selected isolates are listed in Supplementary Tables 3, 4. The mean total length of assembled genomes was 5,037,451 bp, ranging from 4,761,764 to 5,349,454 bp (Supplementary Table 5). The commonly referenced genomes of *S*. 4,5,12:i:- (Australian ref. CP019647 and UK ENA ref LN999997.1) are both at 5.0 Mbp (Dyall-Smith et al., 2017; Mastrorilli et al., 2018), indicating that the assembled genome lengths of our isolates fit well with the genome sizes of other previously reported *S*. 4,5,12:i:-. Based on *in silico* MLST results from Center for Genomic Epidemiology, all selected Thai *S.* 4,5,12:i:- isolates were assigned to ST34.

**FIGURE 1:**
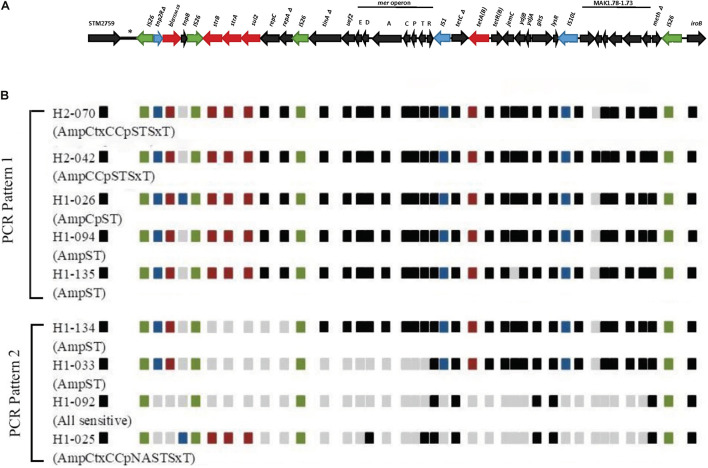
Schematic diagram showing structures and arrangement of relevant antimicrobial resistance genes located between STM2759 and *iroB* gene in selected Thai *S*.4,5,12:i:- isolates. **(A)** Genetic mapping showing resistant region 3 (RR3) between STM2759 and *iroB* gene in the European *S*.4,5,12:i:- reference strain 07-2006 (GenBank: accession number KR856283) based on García et al. (2016), **(B)** Comparison of AMR genes structures and locations within specific region in selected Thai *S*.4,5,12:i:- isolates. Green boxes represent insertion sequence (IS*26*) and blue boxes represent mobile elements other than IS*26*. Resistance genes are indicated by red-colored boxes. Black boxes indicate the presence of genes other than AMR genes while gray boxes show the absence of the genes in these locations that are found in reference strain. Nine isolates are selected here as an example of PCR patterns 1 and 2. Amc, Amoxicillin/Clavulanic acid; Amp, Ampicillin; Ctx, Cefotaxime; C, Chloramphenicol; Cp, Ciprofloxacin; NA, Nalidixic acid; Nor, Norfloxacin; S, Streptomycin; T, Tetracycline; SxT, Sulfamethoxazole/Trimethoprim.

*In silico* comparison of the genome sequences of our selected isolates against strain 07-2006 reference genome (GenBank: accession number KR856283) revealed approximately 16 kb deletion of the *fljAB* operon and surrounding region, with subsequent insertion of multiple resistant genes and metal tolerance genes coupled with a number of mobile genetic elements (MGEs) arranged in a manner similar to the RR3 region found in the European clone (Figure 1). The inserted DNA sequence downstream to STM2759 comprised of *bla_TEM__–1B_*_(_*_*C*_*_)_ determinant encoding β-lactamase enzyme bound by partial resolvase gene (*tnp2R*Δ) and presumptive *tnp* transposase gene, respectively, on either side of resistant gene locus. This composite Tn*2*-like transposon was flanked by a pair of approximately 820 bp long mobile elements equivalent to IS*26* elements. It was followed by the region bearing the genes *aph(6)-Id* (*strB*), *aph(3′′)-Ib* (*strA*) (phosphotransferase genes for streptomycin resistance), *sul2* (gene encoding dihydropteroate synthase enzyme for sulfonamides resistance), *repC* (gene encoding plasmid replication initiation protein), truncated *repA* (gene encoding regulatory protein required for plasmid replication), and another copy of an IS*26* mobile element in sequential arrangement. In addition, the Tn*10*-like DNA segment composing of *tet* operon (*tetC*Δ, *tetB*, and *tetR* genes responsible for tetracycline resistant and tetracycline repressor protein), *jemC*, *ydjB*, *ydjA*, sodium/glutamate symporter gene (*gltS*), and *lysR* genes was flanked by IS*1* and IS*10L* transposases. Downstream to Tn*10*-like transposon included six ORFs with unknown functions, truncated putative DNA modification methylase gene (*meth*) by the fourth copy of IS*26* element insertion at 5′ end of *iroB* gene. Besides, another translocatable unit called Tn*21*-like transposon was located between Tn*2*-like- and Tn*10*-like transposons and comprised of metal tolerance genes such as mercury resistant genes. This genomic context consisted of a copy of IS*26*, subsets of the mercury resistant operon (e.g., *merE*, *merD* for mercury resistant operon regulator, *merA* encoding mercuric reductase enzyme, *merC*, *merP* for mercuric binding protein, *merT* and *merR* genes), putative transposase encoding *tniA* gene, and *urf2* gene with hypothetical function.

A total of 24 AMR determinants belonging to seven different antimicrobial groups were detected in the sequenced genomes (Supplementary Tables 6, 7). From one AMR gene to 14 different AMR genes were observed in each genome sequence. The most common genes identified belonged to antimicrobial groups associated with resistance to aminoglycosides [*aac(6)-Iaa* (100%), *aph(3′′)-Ib* (*strA*) (81.1%), and *aph(6)-Id* (*strB*) (81.1%)], β-lactam and β-lactamase inhibitors [*bla_TEM__–1B_* (84.9%)], sulfonamides [*sul2* (79.2%)], and tetracyclines [*tetB* (71.7%)]. When we examined the phenotypic AMR patterns obtained by disk diffusion and AMR genes observed in the assembled genomes, we identified 18 different AMR phenotypic patterns and 27 genotypic AMR profiles with the most common genotypic combination being *aac(6′)-Iaa*, *aph(3′′)-Ib*, *aph(6)-Id*, *bla_TEM__–1B_*, *sul2*, *tetB* genes being detected in about 38% of the study genomes (20 of 53 isolates) (Table 4). This genotypic profile was most frequently seen in isolates with AmpST phenotype (16 of 20 isolates with this genotype profile) which was also the most prevalent phenotypic AMR pattern in our study. Correlations between AMR phenotype and genotype of tested drugs were calculated from the following formula (Patchanee et al., 2020): [N_(presence of phenotype and genotype)_ + N_(absence of phenotype and genotype__)_]/total N, where N is the number of isolates and total N is 53. Correlation value of 1 indicates the highest match between AMR phenotype and genotype. Correlation values of the tested antibiotic classes are 1 for aminoglycoside, 1 for β-lactam, 1 for tetracycline, 0.98 for sulfonamide and trimethoprim, 0.92 for phenicol, and 0.87 for quinolone.

**TABLE 4:** Phenotypic AMR patterns related to genotypic AMR genes found in selected isolates (*n* = 53).

**Strain ID**	**AMR pattern**	**Aminoglycoside**	**β lactam**	**Fluoroquinolone**	**Phenicol**	**Sulfonamide**	**Tetracycline**	**Trimethoprim**
H1-009	AmcAmpST	*aac(6′)-Iaa*, *aph(6)-Id*, *aph(3′′)-Ib*	*bla_TEM__–1B_*	−	−	*sul2*	*tetB*	−
H1-012	AmpCtxCCpST	*aac(6′)-Iaa*, *aac(3)-IId*, *aph(3′′)-Ib*, *aph(6)-Id*	*bla_CTX__–__M__–__55_*, *bla*_TEM__–1B_	*qnrS1*	*catA2*, *floR*	*sul2*	*tetA*	−
H1-014	AmpCtxCCpST	*aac(6′)-Iaa*, *aac(3)-IId*, *aph(3”)-Ib*, *aph(6)-Id*	*bla_CTX__–__M__–__55_*, *bla_TEM__–1B_*	*qnrS1*	*catA2*, *floR*	*sul2*	*tetA*	−
H1-021	AmpST	*aac(6′)-Iaa*, *aph(3′′)-Ib*, *aph(6)-Id*	*bla_TEM__–1B_*	−	−	*sul2*	*tetB*	−
H1-022	AmpST	*aac(6′)-Iaa*, *aph(3′′)-Ib*, *aph(6)-Id*	*bla_TEM__–1B_*	−	−	*sul2*	*tetB*	−
H1-025	AmpCtxCC pNASTSxT	*aac(6′)-Iaa*, *aac(3)-IIa*, *aph(3′)-Ia*, *aph(3′′)-Ib*, *aph(6)-Id*	*bla_CTX__–__M__–__55_*	−	*catA2, floR*	*sul2*	*tetA*	−
H1-026	AmpCpST	*aac(6′)-Iaa*, *aac(3)-IId*, *aph(3”)-Ib*, *aph(6)-Id*	*bla_CTX__–__M__–__55_*, *bla_TEM__–1B_*	*qnrS1*	*floR*	*sul2*	*tetA*, *tetB*	−
H1-027	AmpST	*aac(6′)-Iaa*, *aph(3′′)-Ib*, *aph(6)-Id*	*bla_TEM__–1B_*	−	−	*sul2*	*tetB*	−
H1-029	AmpCST	*aac(6′)-Iaa*, *aph(3′′)-Ib*, *aph(6)-Id*	*bla_TEM__–1B_*	−	−	*sul2*	*tetB*	−
H1-033	AmpST	*aac(6′)-Iaa*	*bla_TEM__–1B_*	−	−	−	*tetB*	−
H1-036	AmpCSTSxT	*aadA17*, *aac(6′)-Iaa*, *aac(3)-IId*, *aph(6)-Id*, *aph(3′′)-Ib*	*bla_TEM__–1B_*	−	−	*sul2*	*tetB*	−
H1-039	AmcAmpCpS	*aadA17*, *aac(6′)-Iaa*	*bla_TEM__–1C_*	− *	−	−	−	−
H1-050	AmpCtxCCpST	*aac(6′)-Iaa*, *aac(3)-IId*, *aph(3′′)-Ib*, *aph(6)-Id*	*bla_CTX__–__M__–__55_*, *bla_TEM__–1B_*	*qnrS1*	*floR*	*sul2*	*tetA*, *tetB*	−
H1-070	S	*aac(6′)-Iaa*	*–*	−	−	−	−	−
H1-092	All Sensitive	*aac(6′)-Iaa*	−	−	−	−	−	−
H1-094	AmpST	*aac(6′)-Iaa*, *aph(3′′)-Ib*, *aph(6)-Id*	*bla_TEM__–1B_*	−	−	*sul2*	*tetB*	−
H1-096	AmpST	*aac(6′)-Iaa*, *aph(3′′)-Ib*, *aph(6)-Id*	*bla_TEM__–1B_*	−	−	*sul2*	*tetB*	−
H1-099	AmpCtxCCpST	*aac(6′)-Iaa*, *aph(3′′)-Ib*, *aph(6)-Id*	*bla_TEM__–1B_*	−	−	*sul2*	*tetB*	−
H1-102	AmpCSTSxT	*aadA1*, *aadA2*, *aac(6′)-Iaa*, *aph(3′′)-Ib*, *aph(6)-Id*	*bla_TEM__–1B_*	−	*cmlA1, floR*	*sul1*, *sul2*, *sul3*	*tetB*	*dfrA12*
H1-104	AmpCtxST	*aac(6′)-Iaa*, *aph(3′′)-Ib*, *aph(6)-Id*	*bla_TEM__–1B_*	−	−	*sul2*	*tetB*	−
H1-105	AmpST	*aac(6′)-Iaa*, *aph(3′′)-Ib*, *aph(6)-Id*	*bla_*TEM*__–1B_*	−	−	*sul2*	*tetB*	−
H1-107	AmpCtxCpST	*aac(6′)-Iaa*, *aac(3)-IId*, *aph(3′′)-Ib*, *aph(6)-Id*	*bla_CTX__–__M__–__55_*, *bla_TEM__–1B_*	*qnrS1*	−	*sul2*	*tetB*	−
H1-109	AmpCtxCCpST	*aac(6′)-Iaa*, *aph(3′′)-Ib*, *aph(6)-Id*	*bla_CTX__–__M__–__55_*	*qnrS1*	*catA2, floR*	*sul2*	*tetA*	−
H1-111	AmpST	*aac(6′)-Iaa*, *aph(3′′)-Ib*, *aph(6)-Id*	*bla_*rm TEM*__–1B_*	−	−	*sul2*	*tetB*	−
H1-112	AmpST	*aac(6′)-Iaa*, *aph(3′′)-Ib*, *aph(6)-Id*	*bla_TEM__–1B_*	−	−	*sul2*	*tetB*	−
H1-116	AmpCpS	*aadA3*, *aac(6′)-Iaa*, *aph(3′′)-Ib*, *aph(6)-Id*	*bla_TEM__–1B_*	−*	−	*sul2*	−	−
H1-117	AmpST	*aac(6′)-Iaa*, *aph(3′′)-Ib*, *aph(6)-Id*	*bla_TEM__–1B_*	−	−	*sul2*	*tetB*	−
H1-118	AmpST	*aac(6′)-Iaa*, *aph(3′′)-Ib*, *aph(6)-Id*	*bla_TEM__–1B_*	−	−	*sul2*	*tetB*	−
H1-119	AmpS	*aac(6′)-Iaa*, *aph(3′′)-Ib*, *aph(6)-Id*	*bla_TEM__–1B_*	−	−	*sul2*	−	−
H1-120	AmpCSTSxT	*aadA1*, *aadA2*, *aac(6′)-Iaa*, *aph(3′)-Ia*, *aph(3′′)-Ib*, *aph(6)-Id*	*bla_TEM__–1B_*	−	*cmlA1*	*sul2*, *sul3*	*tetA*, *tetB*	*dfrA12*
H1-121	AmpST	*aac(6′)-Iaa*, *aph(3′′)-Ib*, *aph(6)-Id*	*bla_TEM__–1B_*	−	−	*sul2*	*tetB*	−
H1-123	AmpST	*aac(6′)-Iaa*, *aac(3)-IId*, *aph(3′′)-Ib*, *aph(6)-Id*	*bla_TEM__–1B_*	−	−	*sul2*	*tetB*	−
H1-132	AmpS	*aac(6′)-Iaa*, *aph(3′′)-Ib*, *aph(6)-Id*	*bla_TEM__–1B_*	−	−	*sul3*	−	−
H1-134	AmpST	*aadA17*, *aac(6′)-Iaa*, *aac(3)-IId*	*bla_TEM__–1C_*	−	−	−	*tetB*	−
H1-135	AmpST	*aac(6′)-Iaa*, *aph(3′′)-Ib*, *aph(6)-Id*	*bla_TEM__–1B_*	−	−	*sul2*	*tetB*	−
H1-136	AmpST	*aac(6′)-Iaa, aph(3′)-Ia*	*bla_TEM__–1B_*	−	−	−	*tetB*	−
H1-137	AmpCpS	*aac(6′)-Iaa, aac(3)-IId*	*bla_TEM__–1C_*	− **	−	−	−	−
H1-141	CpT	*aac(6′)-Iaa*	*-*	− *	−	−	*tetB*	−
H1-142	AmpST	*aac(6′)-Iaa, aph(3′′)-Ib, aph(6)-Id*	*bla_TEM__–1B_*	−	−	*sul2*	*tetB*	−
H1-143	AmpS	*aadA17*, *aac(6′)-Iaa*, *aac(3)-IId*	*bla_TEM__–1B_*	−	−	−	−	−
H2-001	AmpCST	*aac(6′)-Iaa*, *aac(3)-IId*, *aph(3′′)-Ib*, *aph(6)-Id*	*bla_TEM__–1B_*	−	*catA2, floR*	*sul2*	*tetA*, *tetB*	−
H2-009	AmpCST	*aac(6′)-Iaa*, *aac(3)-IId*, *aph(3′′)-Ib*, *aph(6)-Id*	*bla_TEM__–1B_*	−	*floR*	*sul2*	*tetA*, *tetB*	−
H2-011	AmpS	*aac(6′)-Iaa*, *aph(3′′)-Ib*, *aph(6)-Id*	*bla_TEM__–1B_*	−	−	*sul2*	−	−
H2-012	AmpCpS	*aac(6′)-Iaa*, *aph(3′′)-Ib*, *aph(6)-Id*	*bla_TEM__–1B_*	− *	−	*sul2*	−	−
H2-014	AmpCSTSxT	*aadA2*, *aac(6′)-Iaa*, *aph(3′′)-Ib*, *aph(6)-Id*	*bla_TEM__–1B_*	−	*catA2*	*sul1*, *sul2*	*tetB*	*dfrA32*
H2-017	AmpST	*aac(6′)-Iaa*, *aph(3′′)-Ib*, *aph(6)-Id*	*bla_TEM__–1B_*	−	−	*sul2*	*tetB*	−
H2-020	AmpCCpSTSxT	*aadA2*, *aac(6′)-Iaa*, *aac(3)-IId*, *aph(3′)-Ia*, *aph(3′′)-Ib*, *aph(6)-Id*	*bla_TEM__–1B_*	*qnrS1*	*catA2*	*sul1*, *sul2*	*tetB*	*dfrA32*
H2-030	AmpST	*aac(6′)-Iaa*, *aph(3′′)-Ib*, *aph(6)-Id*	*bla_TEM__–1B_*	−	−	*sul2*	*tetB*	−
H2-038	AmpST	*aac(6′)-Iaa*, *aph(3′′)-Ib*, *aph(6)-Id*	*bla_TEM__–1B_*	−	−	*sul2*	*tetB*	−
H2-042	AmpCCpSTSxT	*aadA2*, *aac(6′)-Iaa*, *aac(3)-IId*, *aph(3′′)-Ib*, *aph(6)-Id*	*bla_TEM__–1B_*	*qnrS1*	*catA2*	*sul1*, *sul2*	*tetB*	*dfrA32*
H2-047	AmpCpS	*aac(6′)-Iaa*, *aph(3′′)-Ib*, *aph(6)-Id*	*bla_TEM__–1B_*	− *	−	*sul2*	−	−
H2-070	AmpCtxCC pSTSxT	*aadA2*, *aac(6′)-Iaa*, *aac(3)-IId*, *aph(3′)-Ia*, *aph(3′′)-Ib*, *aph(6)-Id*	*bla_CTX__–__M__–__55_*, *bla_TEM__–1B_*	*qnrS1*	*catA2*	*sul1*, *sul2*	*tetB*	*dfrA12*
H2-111	ST	*aac(6′)-Iaa*	*bla_TEM__–1B_*	−	−	−	*tetB*	−

Amc, Amoxicillin/Clavulanic acid; Amp, Ampicillin; Ctx, Cefotaxime; C, Chloramphenicol; Cp, Ciprofloxacin; NA, Nalidixic acid; Nor, Norfloxacin; S, Streptomycin; T, Tetracycline; SxT, Sulfamethoxazole/Trimethoprim. Isolates with intermediate resistant were considered as resistant to tested drugs. *Mutation at parC gene (T255S, N395S, S469A, A620T), ** mutation at parC gene (T255S, N395S, S469A, A620T), and parE gene (F238I). T, threonine; S, serine; N, asparagine; A, alanine; F, phenylalanine; I, isoleucine.

## Discussion

To characterize the STM2759 and *iroB* region in Thai *S*. 4,5,12:i:- isolates and to decipher the contribution of this region to MDR phenotypes, we have compared presence and absence of the five target regions (131L, *tniA*, *tetC*Δ, MAK, and 131R) inserted between the two STM2759 and *iroB*, among the Thai and four international *S*. 4,5,12:i:- isolates, characterized the antimicrobial susceptibility phenotypes, and analyzed the genetic basis of AMR in representative Thai *S*. 4,5,12:i:- isolates by WGS. The PCR screening supported the previous report by Huoy et al. (2014) as all of the 108 Thai *S*. 4,5,12:i:- isolates characterized in this study were found to be lacking the genomic region containing genes required for phase 2 flagellin expression and phase inversion with preservation of *iroB.* This is distinct from the isolates of Spanish and U.S. origins in which *iroB* is deleted and 7-kb fragment is inserted, respectively (Soyer et al., 2009). The major PCR pattern (pattern 1) in our study isolates was consistent with that observed in European *S.* 4,5,12:i:- clones isolated from human, food, and animal sources in Germany, Switzerland, and Italy (García et al., 2016). PCR pattern 1 isolates contained the five PCR target regions, suggesting they also harbored RR3 insertions with AMR and metal tolerance genes instead of the *fljAB* operon. In García et al. (2016), 84% of European *S.* 4,5,12:i:- strains with R-type AmpSSuT pattern were positive for all five PCR targets. While in our finding, out of 85 isolates having PCR pattern 1, 81 isolates (95%) were resistant to multiple drugs with R-type AmpST pattern as the majority. These findings suggested that AMR genes inserted into this RR3 likely confer MDR phenotypes. However, the reason for the Spanish and U.S. strains being resistant to a number of antimicrobial drugs even though the target regions were absent remains unclear. The resistant genes could be outside of this specified region or there exist alternative resistance mechanisms.

Based on previous observations, there are a number of mechanisms contributing to the monophasic status of *S*. 4,5,12:i:- variant: (i) deletion of *fljB* gene; (ii) deletions or mutations in *fljA* and *hin* genes (Soyer et al., 2009); and (iii) trapping of *fljAB* promoter containing invertible gene segment in one position with subsequent expression of only *fliC* gene encoding phase 1 flagellin (Zamperini et al., 2007). In this current study, IS*26*-mediated replacement of the *fljAB* operon, *hin* and flanking region by multi-resistant gene clusters and surrounding genetic environment was observed as a causative mechanism for monophasic status of Thai *S*. 4,5,12:i:- isolates similar to monophasic variants from many European countries (Lucarelli et al., 2012; Boland et al., 2015; García et al., 2016) and Australia (Dyall-Smith et al., 2017).

There have been increasing reports on the observation of multiple copies of IS*26* intermingled with various resistance genes among multi-resistant Gram-negative pathogens including STm and its monophasic variant *S*. 4,5,12:i:- (Dionisi et al., 2009; Soyer et al., 2009; Lucarelli et al., 2012; Nigro et al., 2013; Venturini et al., 2013; Doi et al., 2014; Boland et al., 2015; García et al., 2016; Dyall-Smith et al., 2017). IS*26* plays a significant role in mobilization of chromosomal regions containing resistance genes or plasmid-borne or transposon-harboring AMR genes by transposition between distinct genetic areas (Harmer et al., 2014). Following insertions of multiple IS*26* copies at several chromosomal locations, deletion of genetic region in between two adjacent IS*26* copies, e.g., *fljAB* promoter and surrounding genetic environment, occurs by genetic recombination (Boland et al., 2015; He et al., 2015). More importantly, the presence of only a single copy of IS*26* in the chromosome or plasmid predisposed to successive insertion of IS*26* with arrays of resistant genes (Partridge et al., 2018), may lead to emergence of MDR bacterial clones. Moreover, insertion of IS*26* flanking AMR genes bearing composite transposon in bacterial chromosome has also been implicated in the widespread dissemination of AMR genes in monophasic variant (Boland et al., 2015), contributing to selective advantages for the bacteria.

Based on *in silico* MLST results, all selected Thai *S.* 4,5,12:i:- isolates were assigned to ST34. The MLST results from our study were the same with the ST type of *S.* 4,5,12:i:- isolates from other countries such as China, Europe, and Australia (Garcia et al., 2014; Yang et al., 2015; Alicia et al., 2018). However, MLST results of *S.* 4,5,12:i:- human isolates from Malaysia was of ST19 (Ngoi et al., 2018). We have reported here that *S*. 4,5,12:i:- monophasic strains isolated from geographically distant areas, Thailand and Europe, without apparent epidemiological links were observed to have in common the region of resistomes responsible for R-type AmpST, mercury resistant operon, and a number of mobile elements in the specific genomic loci between STM2759 and *iroB*. This could possibly due to the IS*26*-mediated event that could help in mobilization of AMR genes harboring transposons in geographically dispersed enterobacterial populations, and in evolution of multi-resistant pathogens (Reid et al., 2015).

Since genes conferring resistance to antimicrobials are often found co-localizing with metal tolerance genes on the same mobile elements, the issue concerning whether excessive use of metal ions could cross-select for the emergence of antimicrobial resistance is of great interest (Witte, 2013). The possible co-selection of AMR determinants and metal tolerance genes in zoonotic MDR *Salmonella* including *S*. 4,5,12:i:- may suggest selective pressure caused by excessive application of antibiotics or non-antibiotic compounds such as heavy metals with antibiotic activity in the food-producing animals and in animal farming management (Seiler and Berendonk, 2012; Mourao et al., 2015). Although mercury is rarely used in agriculture as pesticide or in clinical settings due to its toxicity, various metals including mercury at certain concentration occur in different natural and polluted environments (Kholodii et al., 1993; Gullberg et al., 2014). Such metal resistance genes carried on the MGEs of environmental bacterial communities can promote the acquisition of antibiotic resistance genes found together on the same MGE from environmental bacteria to clinical pathogens (Pal et al., 2015). Environmental exposure to mercury can potentially act as a driving force for increased antibiotic resistance which has been observed in *E. coli* isolates from different human populations (Skurnik et al., 2010).

Among antibiotic classes, the highest concordance between phenotypic and genotypic AMR characteristics was seen for the tetracycline drugs since all isolates showing resistance to aminoglycoside, β-lactam, and tetracycline. However, discrepancies between phenotypic and genotypic AMR results for other drugs were observed as either the presence of AMR genetic determinants in isolates with corresponding drug susceptibility phenotypes or undetectable AMR genes in isolates showing phenotypic resistance. For example, H1-026 isolate showed the presence of chloramphenicol resistance determinant *floR* gene but expressed chloramphenicol sensitive pattern in phenotypic characterization. In contrast, resistance to ciprofloxacin and nalidixic acid was phenotypically detected despite the apparent absence of quinolone resistance genes in H1-025 isolate. These findings may indicate impaired expression of resistant genes or alternative resistance mechanisms other than known resistance genes, respectively. Non-synonymous mutations in *parC* have also been identified at T255S, N395S, S469A, and A620T (Table 4). Although these changes have not been proven to confer quinolone resistance, all strains with these mutations exhibited resistance to ciprofloxacin, nalidixic acid, or both. Our findings confirmed that AMR testing alone is not sufficient for monitoring of AMR trends and a combination with WGS will better provide a more comprehensive antimicrobial resistance profile.

## Conclusion

The most common R-type AmpST pattern observed in Thai *S*. 4,5,12:i:- isolates have been found associated with the presence of five target regions, between two specific genetic loci replacing the *fljAB* operon. We have demonstrated that simple targeted PCR can be used in under-resourced facilities as a tool to rapidly identify MDR *S.* 4,5,12:i:- isolates, specifically those with R-Type AmpST. Detailed analyses of genome sequences of representative Thai isolates have not only confirmed the AMR phenotypes at high phenotype-genotype correlations but also revealed that IS*26* MGEs, present between STM2759 and *iroB* chromosomal loci, likely mediated insertions of certain AMR genes such as *bla_TEM__–1B_*, *strB-A*, *sul2*, and *tetB* genes as well as mercury tolerance genes. The remaining AMR genes were dispersed throughout the chromosome outside this specific region. The inserted resistance region is similar among *S*. 4,5,12:i:- isolates, within Thailand and also isolates from distant geographic areas with no known epidemiological links, such as European countries. To further investigate the commonality of this inserted resistant region in place of *fljAB* operon and *hin*, additional studies are needed to assess the AMR genetic regions in other clinically important monophasic serovars such as *S.* Typhi (antigenic formula of 9,Vi:a,d:-) and *S.* Enteritidis (antigenic formula of 1,9,12:g,m:-) circulating throughout the world.

## Data Availability Statement

The datasets presented in this study can be found in online repositories. The names of the repository/repositories and accession number(s) can be found below: https://www.ncbi.nlm.nih.gov/bioproject/PRJNA675488.

## Author Contributions

AW and SS-a collaborated in the experimental designs, performed the experiments, analyzed the results, and prepared the manuscript. RO and WW assisted in WGS, data analysis, and manuscript preparation. JC assisted in WGS and sequence curation. SC designed, supervised the experiments and the results analyses, and prepared the manuscript. All authors contributed to the article and approved the submitted version.

## Conflict of Interest

The authors declare that the research was conducted in the absence of any commercial or financial relationships that could be construed as a potential conflict of interest.

## Publisher’s Note

All claims expressed in this article are solely those of the authors and do not necessarily represent those of their affiliated organizations, or those of the publisher, the editors and the reviewers. Any product that may be evaluated in this article, or claim that may be made by its manufacturer, is not guaranteed or endorsed by the publisher.

## Footnotes

1
https://cge.cbs.dtu.dk/services/MLST

2
https://cge.cbs.dtu.dk/services/ResFinder/

3
https://cge.cbs.dtu.dk/services/cge/

4
http://rast.nmpdr.org/

5
http://pubseed.theseed.org/
