# Supplementary material for: Sequence Analyses and Phenotypic Characterization Revealed Multidrug Resistant Gene Insertions in the Genomic Region Encompassing Phase 2 Flagellin Encoding fljAB Genes in Monophasic Variant Salmonella enterica Serovar 4,5,12:i:- Isolates From Various Sources in Thailand
Source: Front Microbiol. 2021 Oct 4;12:720604. doi: 10.3389/fmicb.2021.720604 (PMC8524439; doi:10.3389/fmicb.2021.720604)
Supplement: Supplementary file 1 [file Data_Sheet_1.pdf]

Table S1. List of *S.* 4,5,12:i:- isolates included in the study, their respective sources, sample characters, date of collection, countries, PCR, AMR, and PFGE patterns.

No	Strain	Source	Sample characters	Date of collection (Month/Date/Year)	Country of origin	PCR pattern	AMR pattern	PFGE pattern (27)
1	H1-021	Human	Rectal Swab	2/12/2009	Thailand	1	14	MU-06
2	H1-022	Human	Rectal Swab	2/12/2009	Thailand	1	14	MU-07
3	H1-025	Human	Urine	4/24/2009	Thailand	2	1	MU-10
4	H1-026	Human	Blood	6/23/2009	Thailand	1	12	MU-11
5	H1-027	Human	Stool	5/20/2010	Thailand	1	14	MU-15
6	H1-028	Human	Pus	5/20/2010	Thailand	1	14	MU-07
7	H1-029	Human	Stool	5/25/2010	Thailand	1	11	MU-16
8	H1-030	Human	Stool	5/25/2010	Thailand	1	14	MU-11
9	H1-031	Human	Blood	7/20/2009	Thailand	1	14	MU-11
10	H1-032	Human	Stool	5/25/2010	Thailand	1	14	MU-11
11	H1-033	Human	Stool	5/25/2010	Thailand	2	14	MU-11
12	H1-034	Human	Stool	5/27/2010	Thailand	1	14	MU-11
13	H1-035	Human	Rectal Swab	5/27/2010	Thailand	1	14	MU-11
14	H1-036	Environment	Boot Swab	6/9/2010	Thailand	1	6	MU-11
15	H1-039	Animal	Cloacal Swab	6/9/2010	Thailand	2	8	MU-22
16	H1-091	Human	Stool	1/11/2010	Thailand	1	14	MU-11
17	H1-092	Human	Stool	1/15/2010	Thailand	2	21	MU-11
18	H1-094	Human	Rectal Swab	2/19/2010	Thailand	1	14	MU-25
19	H1-095	Human	Stool	2/23/2010	Thailand	1	14	MU-06
20	H1-096	Human	Stool	3/9/2010	Thailand	1	14	MU-48
21	H1-097	Human	Rectal Swab	3/26/2010	Thailand	1	14	MU-11
22	H1-098	Human	Rectal Swab	4/19/2010	Thailand	1	14	MU-11
23	H1-099	Human	Stool	4/20/2010	Thailand	1	3	MU-06
24	H1-102	Human	Stool	4/30/2010	Thailand	1	6	MU-30
25	H1-103	Human	Rectal Swab	4/30/2010	Thailand	1	14	MU-11
26	H1-104	Human	Rectal Swab	4/30/2010	Thailand	1	10	MU-34
27	H-105	Human	Stool	5/12/2010	Thailand	1	14	MU-35
28	H1-106	Human	Blood	5/12/2010	Thailand	1	14	MU-25
29	H1-107	Human	Stool	5/25/2010	Thailand	1	5	MU-11
30	H1-108	Human	Stool	5/25/2010	Thailand	1	14	MU-11
31	H1-109	Human	Rectal Swab	5/27/2010	Thailand	2	3	MU-11
32	H1-110	Human	Rectal Swab	5/27/2010	Thailand	1	14	MU-11
33	H1-111	Human	Rectal Swab	5/27/2010	Thailand	1	14	MU-39
34	H1-112	Human	Stool	6/9/2010	Thailand	1	14	MU-40
35	H1-113	Human	Stool	6/9/2010	Thailand	1	14	MU-11
36	H1-114	Human	Stool	6/10/2010	Thailand	1	14	MU-11

Table S1. List of *S.* 4,5,12:i:- strains included in the study, their respective sources, sample characters, date of collection, countries, PCR, AMR, and PFGE patterns. (cont.)

No	Strain	Source	Sample character	Date of collection (Month/Date/Year)	Country of origin	PCR pattern	AMR pattern	PFGE pattern (27)
37	H1-115	Human	Stool	6/23/2010	Thailand	1	14	MU-11
38	H1-116	Food	FRM	1/15/2010	Thailand	2	13	MU-41
39	H1-117	Food	FRM	1/15/2010	Thailand	1	14	MU-11
40	H1-118	Environment	NA	1/18/2010	Thailand	1	14	MU-11
41	H1-119	Food	FRM	2/17/2010	Thailand	1	16	MU-11
42	H1-120	Food	FRE	2/22/2010	Thailand	1	6	MU-42
43	H1-121	Animal	NA	3/3/2010	Thailand	1	14	MU-11
44	H1-123	Animal	NA	3/12/2010	Thailand	1	14	MU-22
45	H1-125	Animal	NA	3/22/2010	Thailand	1	14	MU-11
46	H1-126	Animal	NA	3/22/2010	Thailand	1	14	MU-11
47	H1-127	Animal	NA	3/22/2010	Thailand	1	14	MU-11
48	H1-128	Animal	NA	3/22/2010	Thailand	1	14	MU-11
49	H1-129	Animal	NA	3/22/2010	Thailand	1	14	MU-11
50	H1-130	Food	FRE	4/7/2010	Thailand	1	14	MU-11
51	H1-131	Food	FRE	5/21/2010	Thailand	1	14	MU-11
52	H1-132	Animal	NA	6/9/2010	Thailand	1	16	MU-45
53	H1-133	Animal	NA	6/9/2010	Thailand	1	14	MU-11
54	H1-134	Environment	NA	6/9/2010	Thailand	2	14	MU-11
55	H1-135	Food	FRM	6/10/2010	Thailand	1	14	MU-16
56	H1-136	Food	FRE	6/22/2010	Thailand	3	14	MU-49
57	H1-137	Animal	NA	7/7/2010	Thailand	7	13	MU-04
58	H1-138	Animal	NA	7/7/2010	Thailand	1	14	MU-11
59	H1-141	Animal	NA	7/29/2010	Thailand	2	18	MU-11
60	H1-142	Food	FRM	8/2/2010	Thailand	1	14	Untypable
61	H1-143	Environment	NA	10/14/2010	Thailand	2	16	MU-48
62	H1-009	Human	Stool	5/14/2010	Thailand	1	9	MU-01
63	H1-012	Human	Stool	6/2/2010	Thailand	4	3	MU-01
64	H1-014	Human	Stool	6/2/2010	Thailand	4	3	MU-25
65	H1-050	Human	Stool	5/4/2010	Thailand	2	3	MU-28
66	H1-070	Animal	NA	3/22/2010	Thailand	2	20	MU-48
67	H2-001	Animal	Swine carcass	2012	Thailand	2	11	NA
68	H2-004	Animal	Swine carcass	2012	Thailand	2	11	NA
69	H2-008	Animal	Swine carcass	2012	Thailand	2	7	NA
70	H2-009	Food	Pork fat	2012	Thailand	2	11	NA
71	H2-010	Food	Pork skin	2012	Thailand	2	11	NA
72	H2-011	Animal	Swine carcass	2012	Thailand	6	16	NA

Table S1. List of *S.* 4,5,12:i:- strains included in the study, their respective sources, sample characters, date of collection, countries, PCR, AMR, and PFGE patterns. (cont.)

No	Strain	Source	Sample character	Date of collection (Year)	Country of origin	PCR pattern	AMR pattern	PFGE pattern (27)
73	H2-012	Animal	Swine carcass	2012	Thailand	5	13	NA
74	H2-014	Environment	Vehicle, truck	2012	Thailand	1	6	NA
75	H2-017	Animal	Swine carcass	2012	Thailand	1	14	NA
76	H2-019	Environment	Vehicle	2012	Thailand	1	4	NA
77	H2-020	Animal	Swine carcass	2012	Thailand	1	4	NA
78	H2-021	Animal	Swine carcass	2012	Thailand	1	4	NA
79	H2-024	Animal	Swine carcass	2012	Thailand	1	14	NA
80	H2-026	Animal	Swine carcass	2012	Thailand	1	14	NA
81	H2-027	Animal	Swine carcass	2012	Thailand	1	14	NA
82	H2-028	Animal	Swine carcass	2012	Thailand	1	14	NA
83	H2-030	Environment	Vehicle	2012	Thailand	1	14	NA
84	H2-033	Animal	Swine carcass	2012	Thailand	1	12	NA
85	H2-038	Food	Pork kidney	2012	Thailand	1	14	NA
86	H2-039	Food	Pork intestine	2012	Thailand	1	14	NA
87	H2-042	Food	Pork intestine	2012	Thailand	1	4	NA
88	H2-043	Food	Pork heart	2012	Thailand	1	12	NA
89	H2-044	Food	Pork kidney	2012	Thailand	1	4	NA
90	H2-045	Food	Pork intestine	2012	Thailand	1	12	NA
91	H2-046	Food	Pork heart	2012	Thailand	1	12	NA
92	H2-047	Food	Pork liver	2012	Thailand	1	13	NA
93	H2-049	Food	Pork intestine	2012	Thailand	1	14	NA
94	H2-050	Food	Pork heart	2012	Thailand	1	16	NA
95	H2-054	Food	Pork intestine	2012	Thailand	1	14	NA
96	H2-055	Food	Pork liver	2012	Thailand	1	14	NA
97	H2-062	Food	Pork intestine	2012	Thailand	1	12	NA
98	H2-065	Food	Pork liver	2012	Thailand	1	14	NA
99	H2-067	Food	Pork kidney	2012	Thailand	1	14	NA
100	H2-070	Food	Pork liver	2012	Thailand	1	2	NA
101	H2-071	Food	Pork heart	2012	Thailand	1	2	NA
102	H2-072	Food	Pork intestine	2012	Thailand	1	2	NA
103	H2-075	Food	Pork intestine	2012	Thailand	1	14	NA
104	H2-082	Food	Pork intestine	2012	Thailand	1	14	NA
105	H2-089	Food	Pork intestine	2012	Thailand	1	16	NA
106	H2-090	Food	Pork intestine	2012	Thailand	1	14	NA
107	H2-104	Animal	Swine carcass	2012	Thailand	1	12	NA
108	H2-111	Animal	Swine carcass	2012	Thailand	2	19	NA

Table S1. List of *S.* 4,5,12:i:- strains included in the study, their respective sources, sample characters, date of collection, countries, PCR, AMR, and PFGE patterns. (cont.)

No	Strain	Source	Sample character	Date of collection (Year)	Country of origin	PCR pattern	AMR pattern	PFGE pattern (27)
109	S9-204	NA	NA	NA	Spanish	9	6	NA
110	S9-238	NA	NA	NA	Spanish	8	15	NA
111	S9-119	Animal	Poultry	NA	US	9	17	NA
112	S5-526	Human	NA	NA	US	9	20	NA

Abbreviations: FRM = frozen raw meat, FRE = frozen ready-to-eat, NA = not available

PCR pattern: 1 = positive in all five regions (+++++), 2 = positive in all regions except 131L (-++++), 3 = negative in 131L and *tniA* regions and positive in *tetC∆*, MAK and 131R (--+++), 4 = Negative in131L and 131R regions and positive in other three regions (-+++-), 5 = positive in all regions except 131R (++++-), 6 = positive in all regions except *tniA* and131R (+-++-), 7 = negative in all regions except MAK region (---+-), 8 = negative in all regions except *tetC∆* region (--+--), 9 = negative in all regions (-----)

AMR pattern represents the isolates showing resistance to the tested drugs. The isolates presenting intermediate resistance were considered as resistant.

Amp = Ampicillin; Amc = Amoxycillin/Clavulanic acid; Ctx = Cefotaxime; C = Chloramphenicol; Cp = Ciprofloxacin; NA = Nalidixic acid; S = Streptomycin; T = Tetracycline; SxT = Sulfamethoxazole/Trimethoprim

1= AmpCtxCCpNASTSxT
2= AmpCtxCCpSTSxT
3 = AmpCtxCCpST
4 = AmpCCpSTSxT
5 = AmpCtxCpST
6 = AmpCSTSxT
7 = AmpCCpST
8 = AmcAmpCpS
9 = AmcAmpST
10 = AmpCtxST
11 = AmpCST
12 = AmpCpST
13 = AmpCpS
14 = AmpST
15 = CST
16 = AmpS
17 = CpS
18 = CpT
19 = ST
20 = S
21 = All sensitive

Table S2. Primer sets used for identification of five target regions in the study, their expected amplicon sizes, and PCR running conditions (based on previous study (García et al., 2016))

Target region	Primer	Primer Sequence (5' to 3')	Amplicon size (bp)	Temp °C, (time) for		
				Denaturation	Annealing	Extension
**131L**	STM2759	GTTTACACGACTGCCTGAA	2819	95 (5min)	55 (30sec)	72 (3min)
	TEM(2)	GGATAATACCGCACCACATA				
tniA	repAΔ(2)	GCAGGTAGATCACCGGGC	2008	95 (5min)	60 (30sec)	72 (3min)
	tniAΔ(2)	CTCGGCGCCAGGTATACG				
tetCΔ	merR[a]	AACCTGACCATTGGCGTTTTT	1337	95 (5min)	55 (30sec)	72 (3min)
	tetCΔ[a]	GGCATCACTTCTTGGATAGG				
**MAK**	lysR(2)	ACCCGCGGCGAGTAAAGC	1918	95 (5min)	55 (30sec)	72 (3min)
	MAK1.78	GTCCTTCAAAATCTATATCAG G				
**131R**	methΔ	ACTTATTCGCCAGATCAAAGG	1836	95 (5min)	60 (30sec)	72 (3min)
	iroB	CTTCGGCTGCTTTCTGTGC				

Table S3. Selected Thai *S.* 4,5,12:i:- isolates for WGS based on PCR and AMR patterns (total 53 isolates)

PCR pattern / AMR pattern*		1	2	3	4	5	6	7	8	9	Total	Selected from each pattern for WGS
		+ + + ++	- + + + +	- - + + +	- + + + -	+ + + + -	+ - + + -	- - - + -	- - + - -	- - - - -		
**AmpCtxCCpNASTSxT**	**1**	0	1	0	0	0	0	0	0	0	1	1
**AmpCtxCCpSTSxT**	**2**	3	0	0	0	0	0	0	0	0	3	1
**AmpCtxCCpST**	**3**	1	2	0	2	0	0	0	0	0	5	1, 2, 2
**AmpCCpSTSxT**	**4**	5	0	0	0	0	0	0	0	0	5	2
**AmpCtxCpST**	**5**	1	0	0	0	0	0	0	0	0	1	1
**AmpCSTSxT**	**6**	4	0	0	0	0	0	0	0	1	5	4, 0 (S9-204)
**AmpCCpST**	**7**	0	1	0	0	0	0	0	0	0	1	0 (H2-008)
**AmcAmpCpS**	**8**	0	1	0	0	0	0	0	0	0	1	1
**AmcAmpST**	**9**	1	0	0	0	0	0	0	0	0	1	1
**AmpCtxST**	**10**	1	0	0	0	0	0	0	0	0	1	1
**AmpCST**	**11**	1	4	0	0	0	0	0	0	0	5	1, 2
**AmpCpST**	**12**	7	0	0	0	0	0	0	0	0	7	1
**AmpCpS**	**13**	1	1	0	0	1	0	1	0	0	4	1, 1, 1, 1
**AmpST**	**14**	56	2	1	0	0	0	0	0	0	59	17, 2, 1
**CST**	**15**	0	0	0	0	0	0	0	1	0	1	0 (S9-238)
**AmpS**	**16**	4	1	0	0	0	1	0	0	0	6	2, 1, 1

Table S3. Selected Thai *S.* 4,5,12:i:- isolates for WGS based on PCR and AMR patterns (total 53 isolates) (cont.)

PCR pattern / AMR pattern*		1	2	3	4	5	6	7	8	9	Total	Selected from each pattern for WGS
		+ + + ++	- + + + +	- - + + +	- + + + -	+ + + + -	+ - + + -	- - - + -	- - + - -	- - - - -		
**CpS**	**17**	0	0	0	0	0	0	0	0	1	1	0 (S9-119)
**CpT**	**18**	0	1	0	0	0	0	0	0	0	1	1
**ST**	**19**	0	1	0	0	0	0	0	0	0	1	1
**S**	**20**	0	1	0	0	0	0	0	0	1	3	1, 0 (S5-526)
**All sensitive**	**21**	0	1	0	0	0	0	0	0	0	1	1
**Total**		85	17	1	2	1	1	1	1	3	**112**	**53**

Note:*AMR profile represents the isolates showing resistant to the tested drugs. The isolates presenting intermediate resistance were considered as resistant.

Amp = Ampicillin; Amc = Amoxycillin/Clavulanic acid; Ctx = Cefotaxime; C = Chloramphenicol; Cp = Ciprofloxacin; NA = Nalidixic acid; S = Streptomycin; T = Tetracycline; SxT = Sulfamethoxazole/Trimethoprim

S9-204 = *S.* 4,5,12:i:- isolate from Spain, S9-238 = *S.* 4,5,12:i:- isolate from Spain, S9-119 = *S.* 4,5,12:i:- isolate from the US, S5-526 = *S.* 4,5,12:i:- isolate from the US

Since these four isolates were not from Thailand, we excluded them from whole genome sequencing. Red numbers indicate group and numbers selected for WGS.

Table S4. List of whole-genome sequenced *S.* 4,5,12:i:- isolates (n=53 isolates)

No.	Strain	Source	PFGE pattern	PCR pattern	AMR pattern	MLST (ST)
1	H1-009	Human	MU-01	1	AmcAmpST	34
2	H1-012	Human	MU-01	4	AmpCtxCCpST	34
3	H1-014	Human	MU-25	4	AmpCtxCCpST	34
4	H1-021	Human	MU-06	1	AmpST	34
5	H1-022	Human	MU-07	1	AmpST	34
6	H1-025	Human	MU-10	2	AmpCtxCCpNASTSxT	34
7	H1-026	Human	MU-11	1	AmpCpST	34
8	H1-027	Human	MU-15	1	AmpST	34
9	H1-029	Human	MU-16	1	AmpCST	34
10	H1-033	Human	MU-11	2	AmpST	34
11	H1-036	Environment	MU-11	1	AmpCSTSxT	34
12	H1-039	Animal	MU-22	2	AmcAmpCpS	34
13	H1-050	Human	MU-28	2	AmpCtxCCpST	34
14	H1-070	Animal	MU-48	2	S	34
15	H1-092	Human	MU-11	2	All Sensitive	34
16	H1-094	Human	MU-25	1	AmpST	34
17	H1-096	Human	MU-48	1	AmpST	34
18	H1-099	Human	MU-06	1	AmpCtxCCpST	34
19	H1-102	Human	MU-30	1	AmpCSTSxT	34
20	H1-104	Human	MU-34	1	AmpCtxST	34
21	H1-105	Human	MU-35	1	AmpST	34
22	H1-107	Human	MU-11	1	AmpCtxCpST	34
23	H1-109	Human	MU-11	2	AmpCtxCCpST	34
24	H1-111	Human	MU-39	1	AmpST	34
25	H1-112	Human	MU-40	1	AmpST	34
26	H1-116	Food	MU-41	2	AmpCpS	34
27	H1-117	Food	MU-11	1	AmpST	34
28	H1-118	Environment	MU-11	1	AmpST	34
29	H1-119	Food	MU-11	1	AmpS	34
30	H1-120	Food	MU-42	1	AmpCSTSxT	34
31	H1-121	Animal	MU-11	1	AmpST	34
32	H1-123	Animal	MU-22	1	AmpST	34
33	H1-132	Animal	MU-45	1	AmpS	34
34	H1-134	Environment	MU-11	2	AmpST	34
35	H1-135	Food	MU-16	1	AmpST	34
36	H1-136	Food	MU-49	3	AmpST	34
37	H1-137	Animal	MU-04	7	AmpCpS	34
38	H1-141	Animal	MU-11	2	CpT	34
39	H1-142	Food	Untypable	1	AmpST	34
40	H1-143	Environment	MU-48	2	AmpS	34
41	H2-001	Animal	NA	2	AmpCST	34
42	H2-009	Food/Pork fat	NA	2	AmpCST	34
43	H2-011	Animal	NA	6	AmpS	34
44	H2-012	Animal	NA	5	AmpCpS	34
45	H2-014	Environment	NA	1	AmpCSTSxT	34
46	H2-017	Animal	NA	1	AmpST	34
47	H2-020	Animal	NA	1	AmpCCpSTSxT	34
48	H2-030	Environment	NA	1	AmpST	34
49	H2-038	Food/Pork kidney	NA	1	AmpST	34
50	H2-042	Food/Pork intestine	NA	1	AmpCCpSTSxT	34
51	H2-047	Food/Pork liver	NA	1	AmpCpS	34
52	H2-070	Food/Pork liver	NA	1	AmpCtxCCpSTSxT	34
53	H2-111	Animal	NA	2	ST	34

Table S5. General information on WGS outputs of representative *S.* 4,5,12:i:- isolates (n = 53 isolates)

Strain	Accession number	Average coverage	Total length of assembled contigs (bp)	# of contigs	GC%	N50 (bp)	MLST (ST)
H1-009	SAMN16709261	58.0605	4945976	76	52.16	232430	34
H1-012	SAMN16709262	62.2804	5152882	98	52.1	232430	34
H1-014	SAMN16709263	67.1192	5045847	93	52.17	253711	34
H1-021	SAMN16709264	57.0466	5072200	64	52.06	282782	34
H1-022	SAMN16709265	64.5577	4949989	70	52.15	270591	34
H1-025	SAMN16709266	68.7973	5220081	105	51.84	270591	34
H1-026	SAMN16709277	194.571	5134833	91	52.14	232430	34
H1-027	SAMN16709267	67.5272	4981016	67	52.15	270591	34
H1-029	SAMN16709268	55.5444	5073558	85	52.15	270591	34
H1-033	SAMN16709269	72.4322	4978461	79	52.13	270591	34
H1-036	SAMN08387023	102.611	5050035	69	52.12	223309	34
H1-039	SAMN09207900	98.1437	4973795	124	52.17	90145	34
H1-050	SAMN16709278	189.293	5135175	100	52.14	232430	34
H1-070	SAMN08387034	102.061	4907691	68	52.15	221139	34
H1-092	SAMN16709270	73.5717	4920951	63	52.16	282782	34
H1-094	SAMN16709279	183.576	4913366	70	52.17	270591	34
H1-096	SAMN16709280	191.626	4802134	73	52.19	282875	34
H1-099	SAMN16709271	70.9754	4988232	71	52.15	270591	34
H1-102	SAMN16709272	54.9898	5349454	101	51.84	232427	34
H1-104	SAMN16709273	60.7345	5057945	54	52.08	282782	34
H1-105	SAMN16709281	186.388	4978914	67	52.12	282875	34
H1-107	SAMN16709274	62.0196	5029852	74	52.15	270591	34
H1-109	SAMN16709275	62.0366	5088460	98	52.1	163156	34
H1-111	SAMN16709282	195.253	4981026	72	52.15	270591	34
H1-112	SAMN16709276	63.553	4943342	53	52.17	282875	34
H1-116	SAMN08387009	97.6291	5124073	136	51.9	82922	34
H1-117	SAMN08387017	70.4395	4941650	106	52.16	140819	34
H1-118	SAMN08387056	87.5758	4947702	80	52.15	184419	34
H1-119	SAMN08386836	90.5138	4936645	104	52.17	150387	34
H1-120	SAMN08386835	56.9666	5154595	141	51.89	93859	34
H1-121	SAMN08386842	90.3469	4933211	124	52.18	90088	34
H1-123	SAMN08386841	93.5302	4976310	96	52.15	134400	34
H1-132	SAMN08386881	123.156	5064934	91	52.09	157866	34
H1-134	SAMN08387053	90.1776	5038851	112	52.13	113818	34
H1-135	SAMN08386838	52.6834	5016088	111	52.09	105975	34
H1-136	SAMN08386837	73.9892	5022424	93	52.1	113467	34
H1-137	SAMN08386806	107.946	4961582	81	52.12	150132	34
H1-141	SAMN08386809	41.1926	4914631	145	52.17	71967	34
H1-142	SAMN08387039	83.1003	4949967	126	52.17	99178	34
H1-143	SAMN08386800	51.2216	5028045	123	52.11	143053	34
H2-001	SAMN08387030	69.8022	5226175	103	52.07	169496	34
H2-009	SAMN08387031	83.1339	5217517	128	52.07	93227	34
H2-011	SAMN08387042	109.233	4963684	121	52.18	90371	34
H2-012	SAMN08387044	81.535	4961515	115	52.18	104799	34
H2-014	SAMN08386802	60.2283	5222654	175	52.01	61431	34
H2-017	SAMN08386843	82.953	4979180	160	52.17	69161	34
H2-020	SAMN08386808	95.1644	5251545	107	51.94	170770	34
H2-030	SAMN08386818	91.1568	5026405	85	52.07	188044	34
H2-038	SAMN08386822	87.2747	4991874	139	52.17	90088	34
H2-042	SAMN08386825	63.9727	5242870	163	51.98	67828	34
H2-047	SAMN08386827	86.0849	4962358	110	52.19	136566	34
H2-070	SAMN08386829	91.5946	5181602	162	51.89	82678	34
H2-111	SAMN08386831	54.7277	5071622	182	52.09	58984	34

Table S6. AMR genes responsible for resistance to aminoglycoside, β-lactam drugs, and β-lactamase inhibitors found in selected 53 isolates (n = 53 isolates)

**Strain ID**	**STM2759-*iroB* insert (kb)***	**Aminoglycosides**										**β-lactam and β-lactamase inhibitors**		
		aac(6')-Iaa	***aac(3)-IIa***	***aac(3)-IId***	***aph(3')-Ia***	***aph(3'')-Ib***	***aph(6)-Id***	***aadA1***	***aadA2***	***aadA3***	***aadA17***	$bla_{CTX-M-55}$	bla_{TEM-1B}	bla_{TEM-1C}
H1-009	33	+	-	-	-	+	+	-	-	-	-	-	+	-
H1-012	17	+	-	+	-	+	+	-	-	-	-	+	+	-
H1-014	14	+	-	+	-	+	+	-	-	-	-	+	+	-
H1-021	34	+	-	-	-	+	+	-	-	-	-	-	+	-
H1-022	34	+	-	-	-	+	+	-	-	-	-	-	+	-
H1-025	17	+	+	-	+	+	+	-	-	-	-	+	-	-
H1-026	29	+	-	+	-	+	+	-	-	-	-	+	+	-
H1-027	34	+	-	-	-	+	+	-	-	-	-	-	+	-
H1-029	34	+	-	-	-	+	+	-	-	-	-	-	+	-
H1-033	21	+	-	-	-	-	-	-	-	-	-	-	+	-
H1-036	34	+	-	+	-	+	+	-	-	-	+	-	+	-
H1-039	11	+	-	-	-	-	-	-	-	-	+	-	-	+
H1-050	32	+	-	+	-	+	+	-	-	-	-	+	+	-
H1-070	9	+	-	-	-	-	-	-	-	-	-	-	-	-
H1-092	8	+	-	-	-	-	-	-	-	-	-	-	-	-
H1-094	34	+	-	-	-	+	+	-	-	-	-	-	+	-
H1-096	34	+	-	-	-	+	+	-	-	-	-	-	+	-
H1-099	34	+	-	-	-	+	+	-	-	-	-	-	+	-
H1-102	33	+	-	-	-	+	+	+	+	-	-	-	+	-
H1-104	34	+	-	-	-	+	+	-	-	-	-	-	+	-
H1-105	34	+	-	-	-	+	+	-	-	-	-	-	+	-
H1-107	34	+	-	+	-	+	+	-	-	-	-	+	+	-
H1-109	11	+	-	-	-	+	+	-	-	-	-	+	-	-
H1-111	34	+	-	-	-	+	+	-	-	-	-	-	+	-
H1-112	34	+	-	-	-	+	+	-	-	-	-	-	+	-
H1-116	29	+	-	-	-	+	+	-	-	+	-	-	+	-
H1-117	34	+	-	-	-	+	+	-	-	-	-	-	+	-
H1-118	34	+	-	-	-	+	+	-	-	-	-	-	+	-
H1-119	15	+	-	-	-	+	+	-	-	-	-	-	+	-
H1-120	33	+	-	-	+	+	+	+	+	-	-	-	+	-
H1-121	34	+	-	-	-	+	+	-	-	-	-	-	+	-

Table S6. AMR genes responsible for resistance to aminoglycoside, β-lactam drugs, and β-lactamase inhibitors found in selected 53 isolates (n = 53 isolates) (cont.)

Strain ID	STM2759-*iroB* insert (kb)*	Aminoglycosides										β-lactam and β-lactamase inhibitors		
		aac(6')-Iaa	*aac(3)-IIa*	*aac(3)-IId*	*aph(3')-Ia*	*aph(3'')-Ib*	*aph(6)-Id*	*aadA1*	*aadA2*	*aadA3*	*aadA17*	*bla*$_{CTX-M-55}$	*bla*$_{TEM-1B}$	*bla*$_{TEM-1C}$
H1-123	27	+	-	+	-	+	+	-	-	-	-	-	+	-
H1-132	28	+	-	-	-	+	+	-	-	-	-	-	+	-
H1-134	29	+	-	+	-	-	-	-	-	-	+	-	-	+
H1-135	34	+	-	-	-	+	+	-	-	-	-	-	+	-
H1-136	26	+	-	-	+	-	-	-	-	-	-	-	+	-
H1-137	9	+	-	+	-	-	-	-	-	-	-	-	-	+
H1-141	17	+	-	-	-	-	-	-	-	-	-	-	-	-
H1-142	34	+	-	-	-	+	+	-	-	-	-	-	+	-
H1-143	12	+	-	+	-	-	-	-	-	-	+	-	+	-
H2-001	34	+	-	+	-	+	+	-	-	-	-	-	+	-
H2-009	34	+	-	+	-	+	+	-	-	-	-	-	+	-
H2-011	17	+	-	-	-	+	+	-	-	-	-	-	+	-
H2-012	16	+	-	-	-	+	+	-	-	-	-	-	+	-
H2-014	33	+	-	-	-	+	+	-	+	-	-	-	+	-
H2-017	33	+	-	-	-	+	+	-	-	-	-	-	+	-
H2-020	34	+	-	+	+	+	+	-	+	-	-	-	+	-
H2-030	34	+	-	-	-	+	+	-	-	-	-	-	+	-
H2-038	33	+	-	-	-	+	+	-	-	-	-	-	+	-
H2-042	34	+	-	+	-	+	+	-	+	-	-	-	+	-
H2-047	16	+	-	-	-	+	+	-	-	-	-	-	+	-
H2-070	35	+	-	+	+	+	+	-	+	-	-	+	+	-
H2-111	22	+	-	-	-	-	-	-	-	-	-	-	+	-
Total	Total	53	1	15	5	43	43	2	6	1	4	8	45	3
%	%	100%	1.9%	28.3%	9.4%	81.1%	81.1%	3.8%	11.3%	1.9%	7.5%	15.1%	84.9%	5.7%

+ and – indicate presence and absence of gene in the genome as identified by ResFinder. Highlighted cell indicates presence of that gene in the STM2759-*iroB* region. * Insert size as compared to Garcia *et al.* (2016).

Table S7. AMR genes responsible for resistance to quinolones, phenicols, sulfonamides, tetracyclines, and trimethoprim found in selected 53 isolates (n = 53 isolates)

**Strain ID**	**quinolones**	**phenicols**			**sulfonamides**			**tetracyclines**		**trimethoprim**	
	qnrS1	***catA2***	***floR***	***cmlA1***	***sul1***	***sul2***	***sul3***	***tetA***	***tetB***	***dfrA12***	***dfrA32***
H1-009	-	-	-	-	-	+	-	-	+	-	-
H1-012	+	+	+	-	-	+	-	+	-	-	-
H1-014	+	+	+	-	-	+	-	+	-	-	-
H1-021	-	-	-	-	-	+	-	-	+	-	-
H1-022	-	-	-	-	-	+	-	-	+	-	-
H1-025	-	+	+	-	-	+	-	+	-	-	-
H1-026	+	-	+	-	-	+	-	+	+	-	-
H1-027	-	-	-	-	-	+	-	-	+	-	-
H1-029	-	-	-	-	-	+	-	-	+	-	-
H1-033	-	-	-	-	-	-	-	-	+	-	-
H1-036	-	-	-	-	-	+	-	-	+	-	-
H1-039	-	-	-	-	-	-	-	-	-	-	-
H1-050	+	-	+	-	-	+	-	+	+	-	-
H1-070	-	-	-	-	-	-	-	-	-	-	-
H1-092	-	-	-	-	-	-	-	-	-	-	-
H1-094	-	-	-	-	-	+	-	-	+	-	-
H1-096	-	-	-	-	-	+	-	-	+	-	-
H1-099	-	-	-	-	-	+	-	-	+	-	-
H1-102	-	-	+	+	+	+	+	-	+	+	-
H1-104	-	-	-	-	-	+	-	-	+	-	-
H1-105	-	-	-	-	-	+	-	-	+	-	-
H1-107	+	-	-	-	-	+	-	-	+	-	-
H1-109	+	+	+	-	-	+	-	+	-	-	-
H1-111	-	-	-	-	-	+	-	-	+	-	-
H1-112	-	-	-	-	-	+	-	-	+	-	-
H1-116	-	-	-	-	-	+	-	-	-	-	-
H1-117	-	-	-	-	-	+	-	-	+	-	-
H1-118	-	-	-	-	-	+	-	-	+	-	-
H1-119	-	-	-	-	-	+	-	-	-	-	-
H1-120	-	-	-	+	-	+	+	+	+	+	-
H1-121	-	-	-	-	-	+	-	-	+	-	-
H1-123	-	-	-	-	-	+	-	-	+	-	-

Table S7. AMR genes responsible for resistance to quinolones, phenicols, sulfonamides, tetracyclines, and trimethoprim found in selected 53 isolates (n = 53 isolates)

**Strain ID**	**quinolones**	**phenicols**			**sulfonamides**			**tetracyclines**		**trimethoprim**	
	qnrS1	***catA2***	***floR***	***cmlA1***	***sul1***	***sul2***	***sul3***	***tetA***	***tetB***	***dfrA12***	***dfrA32***
H1-132	-	-	-	-	-	+	-	-	-	-	-
H1-134	-	-	-	-	-	-	-	-	+	-	-
H1-135	-	-	-	-	-	+	-	-	+	-	-
H1-136	-	-	-	-	-	-	-	-	+	-	-
H1-137	-	-	-	-	-	-	-	-	-	-	-
H1-141	-	-	-	-	-	-	-	-	+	-	-
H1-142	-	-	-	-	-	+	-	-	+	-	-
H1-143	-	-	-	-	-	-	-	-	-	-	-
H2-001	-	+	+	-	-	+	-	+	+	-	-
H2-009	-	-	+	-	-	+	-	+	+	-	-
H2-011	-	-	-	-	-	+	-	-	-	-	-
H2-012	-	-	-	-	-	+	-	-	-	-	-
H2-014	-	+	-	-	+	+	-	-	+	-	+
H2-017	-	-	-	-	-	+	-	-	+	-	-
H2-020	+	+	-	-	+	+	-	-	+	-	+
H2-030	-	-	-	-	-	+	-	-	+	-	-
H2-038	-	-	-	-	-	+	-	-	+	-	-
H2-042	+	+	-	-	+	+	-	-	+	-	+
H2-047	-	-	-	-	-	+	-	-	-	-	-
H2-070	+	+	-	-	+	+	-	-	+	+	-
H2-111	-	-	-	-	-	-	-	-	+	-	-
Total	9	9	9	2	5	42	3	9	38	3	3
%	17%	17%	17%	3.8%	9.4%	79.2%	5.7%	17%	71.7%	5.7%	5.7%

+ and – indicate presence and absence of gene in the genome as identified by ResFinder. Highlighted cell indicates presence of that gene in the STM2759-*iroB* region.
